# A Case of Labor, with Occlusion of the Vagina and Os-uteri—Requiring Division

**Published:** 1846-12

**Authors:** James P. White

**Affiliations:** Buffalo


*Buffalo, November*, 1846.

---

ART. III. — *A case of Labor, with Occlusion of the Vagina and Os-uteri — requiring division.* Reported by JAMES P. WHITE, M. D.

Oct. 30, 1846,—12 noon. Called with my friend and former pupil, Dr. Charles H. Wilcox, to see Mrs. C., aged eighteen years, in labor with her first child. She had had slight pains during the night, becoming severe in the morning, when Dr. W. was first called to see the patient, and learned that she had been married about two years, but had never before been pregnant. Her menstruation had been regular, until her conception, but scanty and accompanied with severe pain. Conjugal connection had been painful and incomplete for a considerable time after marriage. Dr. Wilcox finding an abnormal state of the organs, desired counsel, when Dr. Hamilton and myself were invited to attend. On examination we found the vagina closed about three inches from its outer orifice by a transverse septum, composed apparently of thick firm membranous bands, extending lower on its rectal or posterior surface than in front, so that the finger could not reach the concavity of the sacrum. The canal was, also, shorter on the right posterior surface than on the left. There seemed a small slit on the back part of this *cul de sac*, which, during a pain, seemed to bring the finger in contact with the *os tincæ*. The patient is of full habit, stature rather below the middle size, nervo-songuineous temperament, pains pretty severe, pulse full and strong, abdomen tender throughout, and external organs very irritable, but well lubricated with mucus. After consultation it was concluded to bleed freely from the arm, give an anodyne, and foment the bowels with hops.

6, P. M. Visited the patient, in consultation with Drs. J. Trowbridge, Hamilton and Wilcox; found she had been more comfortable after the bleeding—that the anodyne had procured some sleep, and that the abdominal tenderness had considerably diminished.

Dr. Trowbridge, after examination, concurs in the diagnosis. On more careful computation from the best data which we could procure in relation to time, we concluded that the full term of utero gestation had not expired, and accordingly prescribed a repetition of the bleeding, an anodyne enema, and fomentations to the abdomen continued. The pulse at this visit were one hundred and twenty, and pretty sharp. The countenance flushed, skin hot and dry.

Oct. 31,—10, P. M. Visited her again with Drs. Hamilton, Wilcox and J. S. Trowbridge. The bleeding the evening previous had been carried to approaching syncope, which, with the morphine, had procured a quiet night and considerable sleep. The pains have returned again to-day, and a repetition of drachm doses of laudanum, in injections, did not arrest the uterine contractions. The presenting mass has descended into the superior strait, and the partition, or substance intervening between the finger and descending tumor is firm, tensely strained, and much less tender than last night. The chink, or crevice discovered yesterday could now be distinctly felt, and ascertained to be about one half inch in its longest diameter, with firm cord-like edges, which were exquisitely tender. I now proceeded by means of the speculum to dilate the *os externum* and canal, and bring completely into view the presenting tumor and the partition across the vagina. During each contraction we could see the water escaping through the crevice above mentioned. The expanded septum or membrane, seemed supported by thick ligamentous bands, or stays running across, with narrow spaces.

Being satisfied that the obstruction was of a character not to be overcome by nature, after consultation, I introduced into the vagina a long curved, probe-pointed bistory, passing it without difficulty into the orifice already mentioned, and made slight nicks toward the left acetabulum, in which direction this crevice was situated; then turning the instrument toward the right foramen ovale, I in like manner scarified pretty freely its edges; turning the cutting edge of the knife now toward the right sacro-iliac junction I made with some difficulty through a thick muscular mass, an incision one-half to three-fourths of an inch in length. The last incision was accompanied with pain, and a slight flow of blood, the other wounds were not followed by any discharge of blood. The pain with an expulsive effort, which immediately followed, thrust the speculum nearly from the vagina. I now arose to give the patient a short rest, when Dr. Hamilton, on introducing his finger, found no difficulty in enlarging the opening already made, to two and one-half, or three inches, and was also gratified to learn that all tenderness had disappeared after this division by the knife.

The woman was now laid back into bed in the hope that nature could complete the process of dilatation. It should here be remarked, that this septum and its edges seemed to form the lower floor of the uterus, and the finger could not detect any neck or orifice to that organ beyond it.

Nov. 1, — 10, A. M. The pains subsided at the time of the operation, and have been slight during the night, but increasing this morning without advancing the head in any considerable degree. The pains produce such extreme agony in the lower part of the abdomen, that she resisted them as much as possible. Hypogastrium tender, and bladder distended. Pulse one hundred and twenty, and compressible, countenance anxious, thirst urgent, bowels not moved since labor commenced. Drew off the water by elevating the head, and retaining it there, whilst a catheter was passed between it and the symphisis pubis. Gave Ol. Ricini ℥ii., continued the fomentations, and waited for the operation of the cathartic, in the hope it would increase uterine action, and overcome the remaining resistance arising from the obstruction of the soft parts.

We met again at 5, P. M., when we found that, although the patient had had considerable uterine contraction, the head had made no progress. Being able now to carry the finger beyond this vaginal position into the cavity of the womb, a more thorough examination was made of the superior strait, which we found diminished in its antero-posterior diameter. We also satisfied ourselves that the fœtus was dead, and as the pains, which were now severe, made little or no impression, we concluded to diminish the head. The bladder being again evacuated, I introduced the perforator into the cavity of the cranium, divided the bones extensively, and removed the larger portion of the cerebral mass. After waiting an hour for the contraction of the uterus, and expulsion of the child, and finding the pains slight, and child not advancing, we administered the secale cornutum. At this time the patient was feverish, mind excited, pulse quick and soft, strength much exhausted. The second dose of ergot increased the pains in force and frequency, but did not advance the child.

9, P. M. The patient is manifestly sinking; slightly delirious, and symptoms indicate convulsions, of which we have been already induced to apprehend danger. Dr. Wilcox now seized the os frontis with a long toothed forceps, one blade extending nearly to the root of the nose, being defended by the finger from injury to the mother, and made firm traction. In the meantime I applied friction with the hand over the abdomen, and administered cordials. After a few pains she was delivered of a full grown male child;—putrefaction having already commenced. The patient was completely delirious; pulse scarcely perceptible; sweating copiously; and with blanched conntenance. Cold sponging, which had been applied to the head for some time, continued, and repeated the cordials. At half-past 10 she became more calm, when we left her with directions to remain perfectly quiet, and avoid every kind of excitement.

Nov. 2,—10, A. M. She has become composed; slept some this morning; pulse reduced; does not remember the delivery, or any thing which occurred during the night.

Nov. 3. Continued to convalesce.

Nov. 13. Found the patient sitting up; is apparently comfortable; has no abdominal soreness; appetite good, and says she is "perfectly well." On making a vaginal examination, found heavy columns, or edges of ligamentous fibres, one and a half inches within the vulva, running antero-posteriorly, and thickest at their rectal attachment. The finger can be passed behind, by the side of those bands into a pouch formed by the vagina, upon the one side, and these folds upon the other. The left *cul de sac* is deeper than the right, being an inch or more in length, and its upper floor, or boundary, is formed apparently of double folds of mucous membrane. Passing the finger between these barriers, upon the pubal surface of the vagina, about two inches farther on, it slipped directly into the womb, without encountering any neck or lip to that organ. On the posterior surface the uterus is also continuous with the vagina, it being impossible to discover more than a thickening or induration of this canal, as indicative of the termination of the one, and the commencement of the other.

In making an attempt to carry the finger up to the promontory of the sacrum, it is arrested, and falls directly into the cavity of the womb, detecting the entrance into the latter merely by a slight irregularity and thickening of the same canal.

The public arch seems low, but we were unable to ascertain the diameter of the superior strait by reason of the adhesion of the posterior lip of the uterus, and the opposing wall of the vagina.

Indeed, the vagina seemed but an expansion of the orifice made in the uterus by the one incision, on the night of the 31st October, and if not a congenital occlusion of the womb, there must have been extensive inflammation and adhesion, of which the most searching inquiries could elicit no intimation.

